# Use-inspired basic research in medical image perception

**DOI:** 10.1186/s41235-016-0019-2

**Published:** 2016-11-14

**Authors:** Jeremy M. Wolfe

**Affiliations:** 1 Ophthalmology & Radiology, Harvard Medical School, 64 Sidney St. Suite 170, Cambridge, MA 02139-4170 USA; 2 Visual Attention Lab, Department of Surgery, Brigham & Women’s Hospital, 64 Sidney St. Suite 170, Cambridge, MA 02139-4170 USA

**Keywords:** False Alarm, Search Task, Breast Cancer Screening, Visual Search Task, Head Compute Tomography

## Abstract

This journal is dedicated to “use-inspired basic research” where a problem in the world shapes the hypotheses for a study in the laboratory. This brief review presents several examples of “use-inspired basic research” in the area of medical image perception. These are cases where the field of radiology raises an interesting issue in visual cognition. Basic research on those issues may then lead to proposals to improve performance on clinical tasks in medical image perception. Of the six examples given here, the first three ask essentially perceptual questions: How can stereopsis improve medical image perception? How shall we assess the tradeoff between radiation dose and image quality? How does the choice of colors change the interpretation of medical images? The second three examples address attentional issues in those aspects of radiology that can be described as visual search problems: Can eye tracking help us understand errors in radiologic search? What happens if the number of targets in an image is unknown? What happens if, as in radiology screening programs, the target of search is very rare?

## Significance

The ever-increasing volume and variety of medical images draws attention to important problems for which the science of visual cognition may have some answers. Moreover, the field of medical image perception raises fundamental scientific questions that have not been asked in visual cognition. This review highlights some of the possibilities for progress that can arise from interactions between the medical and cognitive communities.

## Introduction

Medical images pose many interesting and important perceptual and cognitive questions. How do you know if this irregular mass has changed size or just shape in the time intervening between two images (van Engeland, Snoeren, Karssemeijer, & Hendriks, 2003)? Why are 20–30 % of breast cancers missed in breast cancer screening (Hoff et al., 2012)? Why don’t computer-aided detection systems help as much as one might think they would (Nishikawa et al., 2012)? Though such questions are interesting, they are often quite hard to answer. Manipulating variables in the clinical setting is difficult for obvious ethical and practical reasons. Even arranging to passively collect data in the clinic can be hard. For instance, how would you know if a patient was “correctly” diagnosed? A false negative/miss error might walk out of the clinic, never to be seen again by the experimenter who would never know that an error had occurred. As experimental participants in laboratory tasks, radiologists are a scarce resource. They tend to be extremely busy and they do not tend to volunteer in studies on the promise of $10/h or extra credit in an Introductory Psychology class. (That said, as a group, they tend to be very interested in perceptual questions and, in principle, would like to help.) Collecting the right stimuli is a challenge as well. Researchers who are not, themselves, experts in radiology will need one of those busy radiologists to cull a set of appropriate images for an experiment. A panel of radiologists may be needed to establish gold standard “truth” about those images by consensus. It is, for example, surprisingly hard to get a group of radiologists to agree that there is nothing clinically suspicious in a “normal” chest X-ray. Moreover, the experimenter does not have the control over the location and properties of a target (like a tumor) that would be typical in an experiment where the computer generates the stimuli.

Radiology, the focus of this article, is just one domain of medical image perception. As Elizabeth Krupinski (2010) points out, the field is much broader, especially now that all sorts of images are routinely digitized, including, for example, the glass slides that pathologists once viewed exclusively under a microscope or photographs of the retina in ophthalmology. The dermatologist who is evaluating a skin lesion might be halfway across the globe, reading an image as part of a telemedicine practice. All of these modalities create stimuli that could, in principle, be used in medical image perception studies. In answering questions in these or other realms of medical image perception, we might find practical constraints similar to those, mentioned above, for radiology.

In this article, I will describe six examples of medical image perception research. Most, but not all of these are projects where our lab has made a contribution. The first three examples are perceptual: dealing with the use of stereopsis in mammography; the effects of different choices for colorizing images; and the perceptual consequences of reducing the dose of radiation in an exam. The last three examples deal with some attentional aspects of medical image perception. What can eye tracking tell us about errors in the reading of medical images? How does search behavior change if there is more than one possible target? What are effects of searching for very rare targets like cancer in a screening population? No one should mistake this for a comprehensive survey. For that the reader could consult *The Handbook of Medical Image Perception and Techniques* (Krupinski & Samei, 2010). The goal here is to entice cognitive researchers to think about trying some “use-inspired, basic research” (Stokes, 1997) in the domain of medical image perception. Publishing such use-inspired, basic research is the mission of this journal.

## Review

### Example 1: stereopsis in mammography

We start with this example because it is one of the rare cases (so far) where the research resulted in a new methodology being approved by the US Food and Drug Administration and, thus, made commercially available. One of the fundamental problems with medical images is that they are two-dimensional (2D) representations of three-dimensional (3D) structures. Of course, this is a central problem in visual perception more generally. The images on our retinae are 2D and from those 2D images we need to infer a 3D world. To this end, we make use of a long list of depth cues, as described in any textbook on Perception. These cues play a role in rendering medical images, as when the structures are shaded as though illuminated by a light source.

**Fig. 1:**
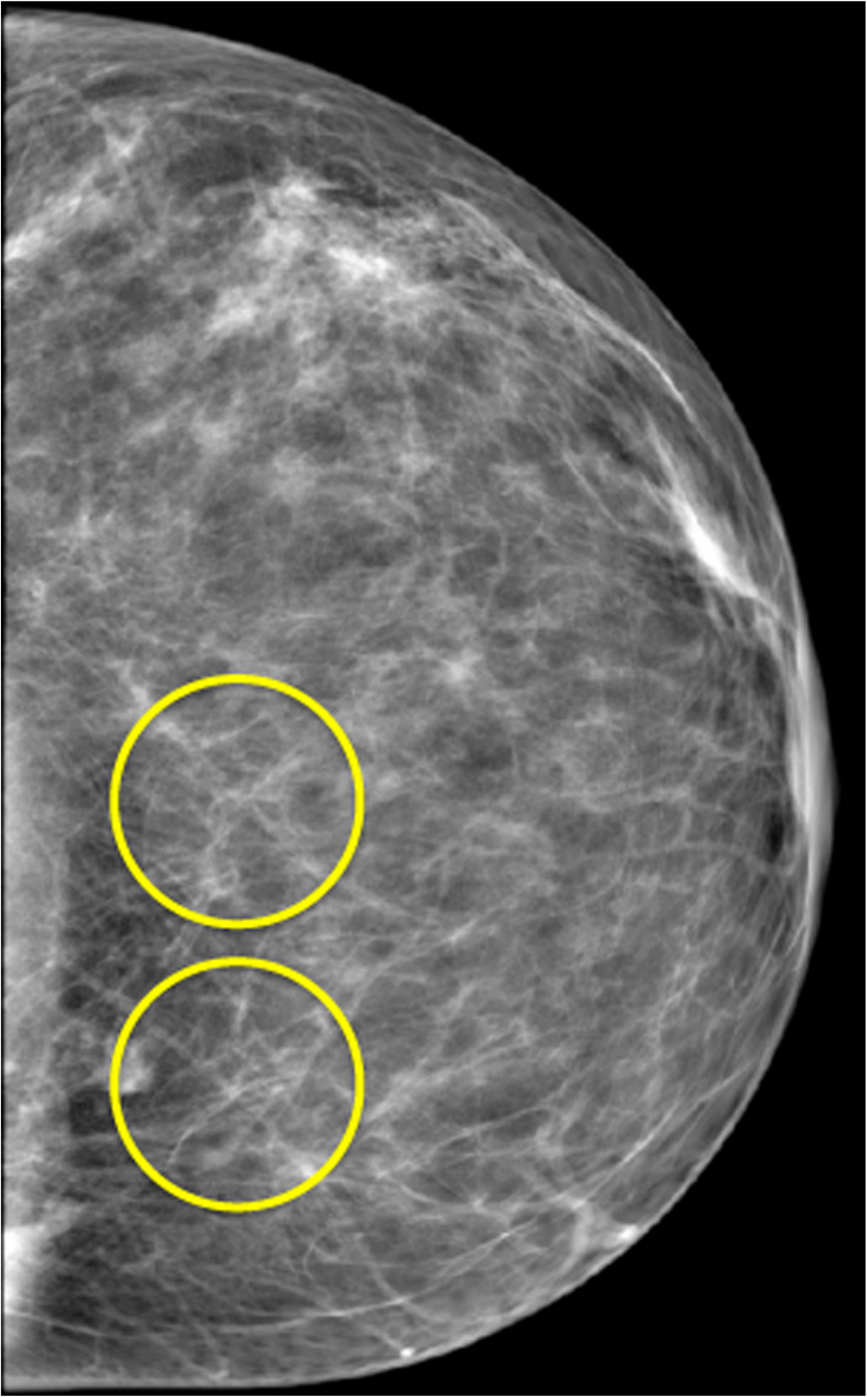
A standard mammogram. Are the intersections in the two *circles* actual structures or the accidental superposition of filaments at different depths in the 3D volume of the breast?

Binocular stereopsis is a depth cue with the potential to be very useful in some medical image domains. Consider the mammogram in Fig. 1.

At the center of each of the yellow circles are radial structures. Those could be clinically significant, if they are real structures in the breast but, because the image collapses in 2D information about the 3D volume of the breast, we do not know if what looks like a radial structure might not just be filaments at different depths, superimposed in the flat image.

Binocular stereopsis is very good at disambiguating such situations. The slight differences between the images in the two eyes can be used to recover the relative position in depth of elements like this (Howard & Rogers, 1995, 2001). Imagine looking up into the branches of a bare tree. With an unmoving head and one eye, it can be quite difficult to decide if one twig is in front of another. With two eyes (and a normal binocular visual system) (Sacks, 2006), the twigs are seen as a much less ambiguous 3D structure.

The thought that this might be useful in medical image perception arose as soon as X-rays appeared. Stereopsis was described by Wheatstone (1838) and Brewster (1841) in the first half of the 19th century, ushering in a consumer craze for stereoscopic photographs. Once X-rays were described by Röentgen in 1895, Thomson (1896) almost immediately suggested that stereoscopic X-rays might be useful. David Getty and his colleagues (Getty, D’Orsi, & Pickett, 2008; Getty & Green, 2007) worked for many years to apply the basic science of stereopsis to the clinical setting of breast cancer screening. This effort culminated in a clinical trial showing measurable advantages over 2D mammography (D’Orsi et al., 2013) and Stereoscopic Digital Mammography is now an approved clinical tool.

Interestingly, Stereoscopic Digital Mammography has not swept the world of breast cancer screening. Instead, the popular new technology is Digital Breast Tomography (DBT). DBT is a bit like a computed tomography (CT) scan of the breast. It creates a stake of virtual slices through the breast and the radiologist scrolls through them. The stack of slices also serves to disambiguate the 2D image. We will return to some of the perceptual issues raised by these methods in Example 4, later in this paper. Nevertheless, Stereoscopic Digital Mammography is an example of how the interaction of basic vision science and a clinical problem can lead to a new clinical tool.

### Example 2: color maps

**Fig. 2:**
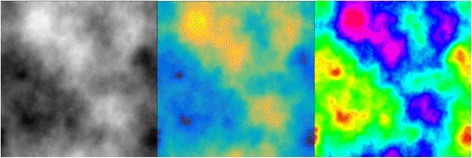
Three ways to present the same set of data in color

Mammograms are typically rendered in grayscale but, as medical images are increasingly digital in format, the options for manipulating and reformatting those images become endless. Consider color. The image is just a representation of a set of numbers. An X-ray does not have “true” colors. The color presented to the observer is a mapping of the set of numbers into some set of colors. The choice of color can alter the story the image seems to tell. Figure 2 is an artificial illustration of this point. The data underpinning these images are generated as random 1/f-sq noise which is a rough approximation of breast or lung tissue. In the first panel, signal strength is mapped to a grayscale. The second and third panels show two color maps of the same data. There is no “right” answer here since the image is just a patch of computer-generated noise, but it should be clear that the different maps tell different stories. Rogowitz and Treinish (1998) argue that the categorical nature of human color perception can give rise to “wrong” or misleading answers. The underlying data in Fig. 2 are continuous but, especially in the third panel, we are inclined to see the red and blue regions as distinct “figures” on a background. If those data values indicate something categorical like bone or air or cancer, that is fine. If they are converting continuous data into artificial “things,” that might be misleading.

### Example 3: dose reduction

**Fig. 3:**
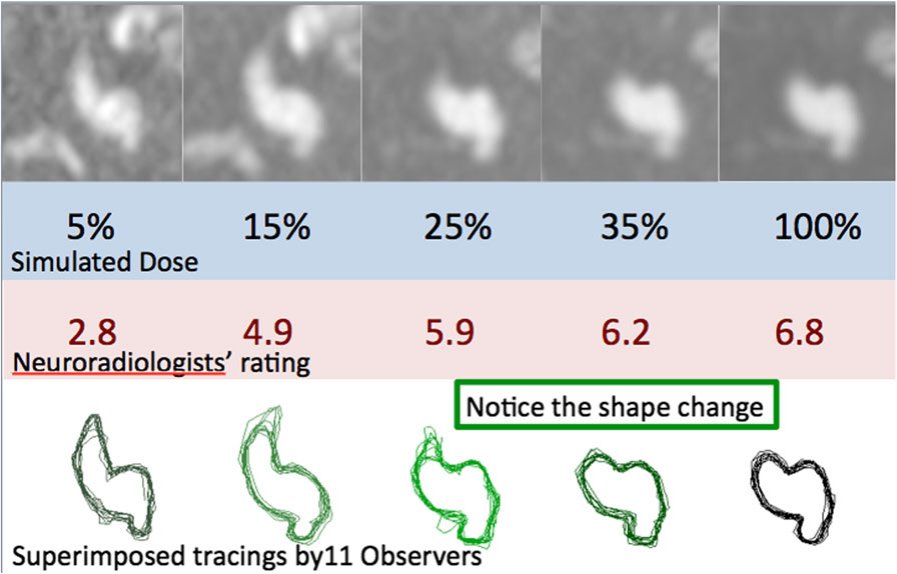
Effects of simulated dose reduction on the ability to trace the shape of an aneurysm in a head *CT image*

One of the problems faced by the researchers who developed stereoscopic mammography was that you need two images. Simply taking two pictures would double the dose of X-rays and that is not desirable. Radiologists often face competing desires to have the best images possible and to have the lowest dose of ionizing radiation possible (e.g. De Zordo et al., 2012; Moscariello et al., 2011; Summers, 2010). Lowering the dose increases the noise in the image, while increasing the dose increases the possibility of harming the patient (e.g. by inducing cancer). This tension sets the stage for some use-inspired basic-research. How do you decide if an image is good enough? The top row of Fig. 3 shows an example of simulating different doses in a head CT. The simulated dose is shown as a percentage in row 2.

The top row of the figure shows a small part of the CT containing the image of an aneurysm. We asked radiologists to rate image quality (row 3) and to trace the aneurysm (row 4). Clearly, the shape and variability of the aneurysm outlines change with dose level. At what dose is the image good enough to do the task? The third row shows that neuroradiologists’ ratings of the image quality decline with dose. Such ratings are imperfectly correlated with formal, computational methods that analyze signal to noise ratios. As with photographs, these computational measures have had difficulty evaluating the impact of factors like clutter that influence human opinions and performance within those scenes (Baldassi, Megna, & Burr, 2005; Rosenholtz, Li, & Nakano, 2007; Zelinsky & Yu, 2015). Moreover, a clinician’s introspective opinion about whether an image is “good enough” may not be linearly related to their ability to do the task at hand. In a pilot study in our lab, for example, radiologists’ ratings of the quality of the head CT declined smoothly with reduction in dose but their performance on a stroke detection task was closer to a step function. The clinicians had good performance using stimuli that they declared to be unacceptable. Thinking about the workflow in a radiology practice, if the image is actually unacceptable, it would be useful to know at the moment it was acquired so that the patient could be rescanned immediately and would not need to return for another round of imaging later. Making this assessment in an automated manner will require a better understanding of the function(s) relating performance to perceived quality and/or physical measures of the image and, again, provide a place for use-inspired basic research that can subsequently inform more strictly applied work.

### Example 4: eye tracking and errors

For the last three examples in this tour of medical image perception, we will consider medical images as visual search stimuli. How do clinicians find—or fail to find—what they are looking for in an image? Medical image perception research has made good use of the methods of basic vision science in trying to answer questions of this sort. Eye movement tracking is one good example. In radiology, eye tracking has been used to develop an influential taxonomy of sources of error in medical image perception. The classic formulation (Kundel, Nodine, & Carmody, 1978) divides errors into three categories based on eye tracking analysis. There are “search” errors in which the target is never fixated. “Recognition” errors are said to occur when the eyes land on the target but only briefly (less than about half a second). Finally, in “decision” errors, the observer fails to respond to a target, even after extensive scrutiny of the target. These errors are traditionally thought to occur with roughly equal frequency in a variety of domains (e.g. bone (Hu, Kundel, Nodine, Krupinski, & Toto, 1994), breast (Krupinski, 1996), and lung (Kundel, Nodine, & Krupinski, 1989)).

It is possible that the pattern of errors may be changing as the technology changes. The older eye tracking data were collected as observers looked at 2D images. As was discussed in Example 1, single images of the breast or chest are now being replaced or supplemented by stacks of many “slices” that, taken together, form a 3D volume of image data. How do people search through a 3D volume of images? Note that this is quite different from asking how people search in the everyday 3D world. A radiologist is examining a 2D slice of the volume at each instant (the XY plane) while scrolling through the slices (the Z- or depth axis). Thus, the eyes can now be tracked in XYZ, using the eye tracker to monitor X & Y and using the slice number as a measure of Z. When we tracked the eye movements of radiologists reading these novel stimuli, we found that they fell into two categories—at least when looking for signs of lung cancer in a chest CT composed of between one and 300 slices. Some radiologists were “drillers,” holding their eyes in a small region of XY while moving through the depth of the volume. Having gone through all of the images, they then moved their eyes to a different neighborhood in XY and again scrolled through the volume. Drillers made multiple passes through the entire stack of images. In contrast, “scanners” tended to make many fewer passes through the stack; perhaps just one. Scanners look all over the XY plane before moving much more slowly through Z.

Our data are not adequate to determine whether drilling or scanning is the superior method, but there is an indication in the data that search errors account for a larger portion of missed items in the 3D presentation compared to 2D. It could be that observers develop an inflated idea about how much of the volume has been examined. It may be harder to keep track of what has been searched in 3D than in 2D. Moreover, the great advantage of the stack of 3D slices in the breast and in the lung is that these images reduce the ambiguity of findings. Thus, decision errors might go down as search errors rise. The difficulty in attracting sufficient numbers of expert observers makes this another venue for use-inspired basic research. We can create artificial search tasks in which novices look for targets in slices through 3D noise. We can then instruct them to drill or scan in an effort to see if one method is inherently superior as we parametrically vary the stimulus. Once the basic research delivers up an answer, we can return to clinicians with a study of manageable length.

### Example 5: satisfaction of search and its relatives

In typical laboratory visual search tasks, there is a target item that is either present or absent. Real world situations like those in radiology have raised a set of otherwise ignored questions that arise when the number of targets in an image is not known. For example, it turns out that if there is more than one target in an image, finding target A can make it less likely that target B will be reported than if only target B had been present. This phenomenon was dubbed “satisfaction of search (SoS, Tuddenham, 1962)” because it was thought that finding the first target “satisfied” the observer. Having found something, the observer abandoned search before finding everything. Berbaum et al. (1991) showed that this idea of an early exit from search is not, in fact, correct, but the name has stuck, though Adamo, Cain, and Mitroff (2013) have suggested “Subsequent Search Misses (SSM)” as a more theory-neutral term (Cain, Adamo, & Mitroff, 2013). Berbaum has pioneered the study of SoS in medical image perception (for a review, see Berbaum, Franken, Caldwell, & Shartz, 2010). Recently, the topic has been taken up with non-medical images (Cain, Dunsmoor, LaBar, & Mitroff, 2011; Fleck, Samei, & Mitroff, 2010), in part because the phenomenon is clearly related to a number of topics that have been of interest to the basic visual cognition community. For instance, one type of SoS error occurs when an observer finds a target and then misses another target of a different variety. This is important in radiology because a radiologist is expected to keep an eye out for so-called “incidental findings,” targets of clinical significance that may not have been the primary object of search (Lumbreras, Donat, & Hernández-Aguado, 2010). If you are looking for lung cancer, you should also report a broken rib, if it happens to be present. To visual cognition researchers, this sounds very similar to the phenomenon of “inattentional blindness” (Mack & Rock, 1998) in which perceptually striking stimuli are seemingly not seen. At the very least, they are not reported. The most famous example of this phenomenon is Simons and Chabris' (1999) experiment in which observers, monitoring a ball game, often fail to notice an actor in a gorilla suit, wandering through the middle of that game. In an homage to that gorilla, we placed an image of a gorilla across five slices in one of the stacks of chest CT images in the driller/scanner experiment, described above. Only four out of 24 radiologists reported this particular, odd incidental finding (Drew, Vo, & Wolfe, 2013). Our radiologists were not “bad” radiologists. They were human radiologists, subject to the same constraints as human observers performing less important tasks.

In the gorilla experiment, radiologists were looking for small, round, white lung nodules and missed a big, irregular, black gorilla. In other SoS experiments, the found and missed targets are of the same kind. Observers fail to report the second nodule or the second letter “T” in a search for “T” among “L”s (Fleck et al., 2010). As Cain et al. (2013) point out, SoS (or SSM) errors most likely arise from multiple causes. In some cases, finding the first target may deplete resources that would otherwise help find subsequent targets (Adamo et al., 2013; Cain & Mitroff, 2013). In some cases (like the gorilla case), properties of the first target guide attention away from a second target. Thus, finding a light-colored T makes it less likely that an observer will find a dark T (Fleck et al., 2010). In some cases, the original idea—that observers simply abandon search too quickly—appears to contribute to SoS errors (Cain, Vul, Clark, & Mitroff, 2012).

These errors may be connected more generally to optimal foraging behavior by humans and other animals. In many situations, it is not in a searcher’s best interest to find every target. When picking blueberries off of a bush, an “optimal” forager should leave for the next bush when the yield from the current bush falls below the average yield for the field of bushes. This rule is formalized as the “marginal value theorem” (Charnov, 1976) and the rule describes a wide range of animal foraging behavior (Stephens, Brown, & Ydenberg, 2007; Stephens & Krebs, 1986). It also describes what humans do in a simple, lab analog of berry picking (Wolfe, 2013). However, this behavior could be decidedly non-optimal in a radiology clinic if a radiologist abandoned the search for metastases in one set of images because the yield was lower than the average from other cases.

In the clinic, the situation is somewhat different from berry picking. Rather than the many, many targets of the blueberry patch, there may only be a few. Modifications of optimal foraging theory like the “potential value” theory (McNamara & Houston, 1985) take this into account, proposing that the observer will move to the next patch or case when the expected rate of target acquisition (rather than the observed rate) falls below the average rate (Ehinger & Wolfe, 2016). The expected rate can be calculated even when the observer is not currently finding anything. It remains to be seen if the foraging rules that may have served us well as hunter-gatherers cause us problems in more modern tasks like those found in medical imaging perception.

### Example 6: the prevalence problem

Finally, consider the problem of target prevalence. In our work, this is a clear example of “use-inspired, basic research” (Stokes, 1997). Why study searches where targets appear on only 1–2 % of trials? Because we ask airport screeners, radiologists, and others to do exactly that: to find important targets that are very rare. Taking breast cancer as an example, there are likely to be three or four cancers for every 1000 women screened in North America (Gur et al., 2004; Lee, Bhargavan-Chatfield, Burnside, Nagy, & Sickles, 2016). With a target prevalence of 0.3–0.4 %, screening mammography is a different type of search task from the standard visual search task in the lab where targets would be present on 50 % of trials (Wolfe, 1998) or even 100 % if the task is one where observers are asked to localize the target or to make a judgment about that target (e.g. what is the orientation of the line inside a square target that is present on each trial? (Theeuwes, 1992)). Do dramatic differences in target prevalence affect our ability to effectively search for that target? The vigilance literature suggests that the answer would be “yes; rare items are likely to be found less often” (Baddeley & Colquhoun, 1969; Colquhoun & Baddeley, 1967) but in those experiments, observers are looking for transient stimuli. In screening tasks, experts are examining complex images that remain present until they choose to move on to the next image.

Studying low prevalence search tasks presents some challenges. In order to assess performance at low prevalence with adequate statistical power, an experiment must consist of many trials in order to get even a handful of target-present trials. Asking expert radiologists to read thousands of cases for an experiment on low prevalence is simply not practical. A radiologist might read thousands of cases in a year, not in an afternoon.

**Fig. 4:**
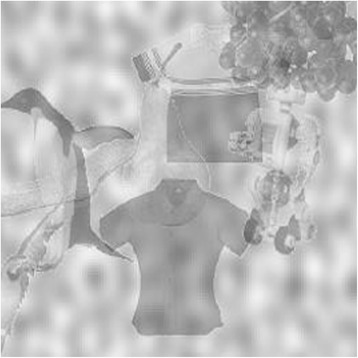
Sample from Wolfe et al. (2005)

Here is where use-inspired, basic research becomes important. The fundamental question—How does target prevalence influence visual search performance?—can be brought into the lab and studied with the usual tools of Experimental Psychology using the usual non-experts as observers. We tested non-expert observers using stimuli like those shown in Fig. 4.

**Fig. 5:**
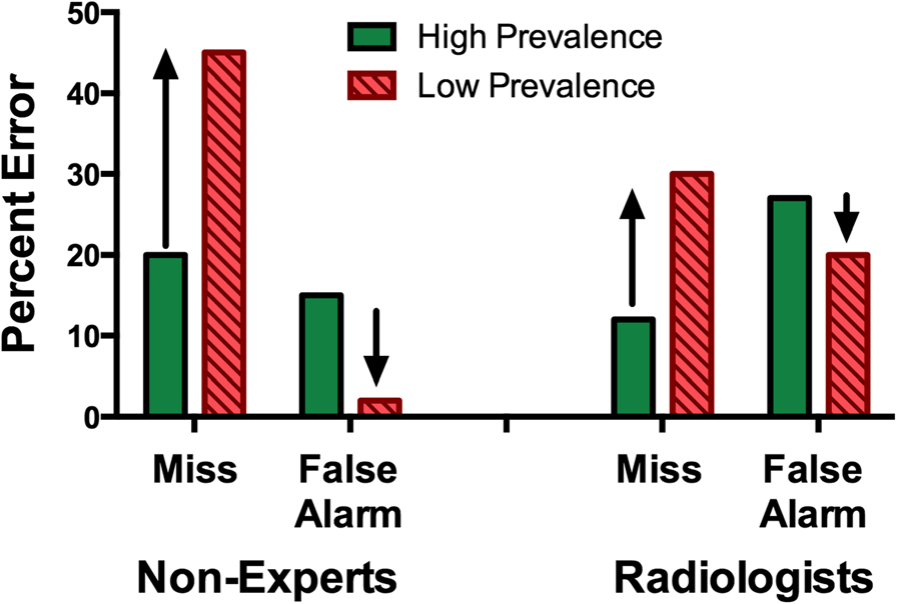
Comparison of prevalence effects in non-expert and radiologist observer populations. In both cases, miss error rates rise at low prevalence while false alarms fall. Non-expert data are replotted from Wolfe et al. (2005). Radiologist data are replotted from Evans, Birdwell, and Wolfe (2013). Differences are statistically significant, as detailed in the original papers

Although our observers were searching for tools, not breast cancer, the stimuli were designed to capture some of the ambiguity and overlapping structure characteristic of X-ray images in radiology and of luggage in baggage screening (another low prevalence search task). With these observers, we could collect 2000 trials at low (2 %) prevalence and a few hundred trials at high (50 %) prevalence. We found the results shown on the left side of Fig. 5. Miss errors (false negatives) were significantly higher at low prevalence. False alarms (false positives) were reduced at low prevalence (Wolfe, Horowitz, & Kenner, 2005). This tradeoff of error type is characteristic of a “criterion shift” in the terms of signal detection theory (Macmillan & Creelman, 2005). In subsequent work, we argued that criterion shifts were an important piece of our prevalence effects, though not the whole story (Wolfe & VanWert, 2010). For instance, we showed that the criterion shift goes the other way when prevalence becomes very high (Wolfe & VanWert, 2010). At high target prevalence, our observers made more false alarm errors and fewer misses. Of course, in screening for cancer or for threats at the airport, misses and false alarms do not have the same importance. The cost of a higher rate of false negative errors is not easily outweighed by the benefits of fewer false positives.

So, low prevalence increases miss errors in the lab with non-experts. Does this prevalence effect occur with experts? We could not ask radiologists to read thousands of images in the lab, but we could and did ask them to read 100 cases; 50 with cancer and 50 without. That was the high prevalence condition. For the low prevalence arm of our study, we added those 100 cases into the normal workflow at our hospital (with informed consent from the radiologists and appropriate safeguards, of course). The 100 cases were spread over many months so they had minimal impact on the low baseline prevalence of cancer in the mammography program. The basic result is shown on the right of Fig. 5, replotted from Evans, Birdwell, and Wolfe (2013). The pattern is the same. At low prevalence, miss error rates are more than double the high prevalence miss error rate while false alarm errors are somewhat lower. Similar effects are seen with cytologists reading cervical cancer screening tests (Evans, Tambouret, Wilbur, Evered, & Wolfe, 2011) and with newly-trained airport screeners (Wolfe, Brunelli, Rubinstein, & Horowitz, 2013).

We used fairly subtle cancers in the study with radiologists. Still, the 30 % error rate, found when we inserted cases in to the normal workflow, falls in the 20–30 % range that is estimated for the percentage of cancers that are missed in screening (e.g. Hoff et al., 2011). This suggests that, perhaps, half of the errors in screening might be due to the low prevalence problem and that “curing” the problem would be a valuable goal.

Again, we will need use-inspired, basic research if we want to try to “cure” the prevalence effect because we cannot cavalierly manipulate clinical practice in an effort to shift radiologists’ decision criteria. Before suggesting an intervention in the clinic, we would want evidence that it has worked in the lab. We have tried a range of interventions in the lab (mostly using baggage X-rays in work collaborating with the US Department of Homeland Security). Most of them did not work (Wolfe et al., 2007). However, giving observers an initial burst of high prevalence practice with full feedback seemed to help when they then went on to do a block of trials at low prevalence without feedback (in the clinic or at the airport, of course, the expert gets only partial feedback, at best). We have not had the chance to implement this in a radiology setting but there is some evidence that this intervention is useful in the airport setting (Wolfe et al., 2013).

**Fig. 6:**
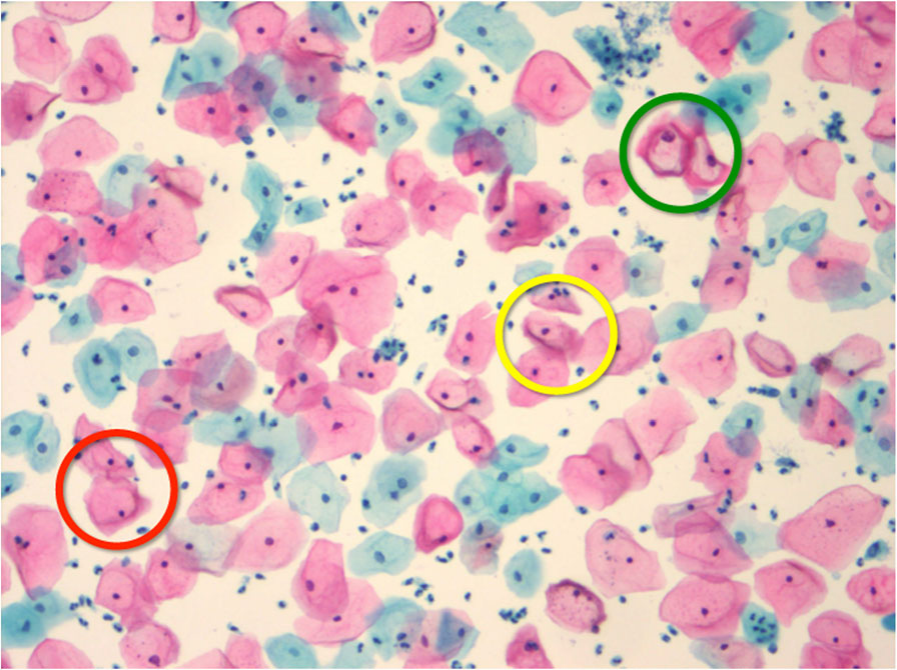
A single “trial” in the search for cervical cancer. An actual case could have many thousands of cells on it. This is a small section. Only the *green circled* cells were actually marked as abnormal by a clinician. The other marks are for purposes of discussion. See text for details

The prevalence problem, with its criterion shift, is just one of many medical image questions that seem like natural candidates for signal detection analyses. Medical images present some unusual challenges for signal detection—at least as it is typically deployed in vision research. In a standard task, vision researchers might ask their observers to make a single two-alternative, forced choice decision or, perhaps, to give a single rating of an image. From those responses, we may calculate proportions of hits, miss, true negatives, and false alarms. From there, we can derive measures of performance such as d’ or the area under the ROC curve. Now, consider the situation in which we ask a hypothetical cytopathologist to read the cervical cancer test (Pap smear) in Fig. 6.

The cytologist might correctly identify the cells, marked by the green circle, as abnormal. She might miss the locus marked by a red circle and she might mark the area in the yellow circle, even though it is not abnormal (Note: only the green circled cells are actually abnormal as determined by a pathologist). Thus, on this one “trial,” we would have a hit, a miss, and a false alarm. A separate branch of signal detection theory has been developed to handle such “free response” tasks. Here, FROC (Free Response Operating Characteristic) curves or JAFROC curves (Jackknife Free Response Operating Characteristic (Chakraborty, 2006)) replace standard ROCs. These plots have the number of false alarms on the x-axis rather than the proportion of errors. This is not the place for a tutorial on these methods but they are extensively reviewed elsewhere (e.g. Burgess, 2010; Chakraborty, 2013). As an aside, it is worth noting that the medical world has a different vocabulary for signal detection analyses. Most notably, the term “sensitivity” typically refers to d’ in the psychological signal detection literature. It refers to the hit/true positive rate in the medical literature. “Specificity” is the true negative rate. The axes of ROCs in the medical literature are typically labeled as “sensitivity” and “1-specificity.”

## Conclusions

### Where do great scientific questions come from?

There are many routes to discovering interesting and important questions in basic vision science. Those of us who do basic research in vision and attention typically expect the next important question to emerge from our thoughts about the last important results, building a cumulative edifice of science. The examples briefly discussed here illustrate another path; not better or worse, just different. This is the path of “use-inspired, basic research” where changes in the world outside of the science raise a set of worthwhile questions that might not have otherwise occurred to us. Often this is because the outside world delivers new stimuli for our consideration. Thus, the effect of prevalence in search becomes an interesting basic research question because modern society has invented low prevalence screening tasks that are important to our health and security. Studying the basic science of prevalence effects ultimately gives us fundamental facts about search behavior in general, as well as a way to help identify and, perhaps, ameliorate a source of errors in these socially important, real-world tasks.

The mission of this journal is to foster research of this sort in the cognitive and behavioral sciences. The “use-inspired” mindset can produce valuable lines of basic research. Moreover, by encouraging and publishing a body of use-inspired research, *Cognitive Research: Principles and Implications* can do its part to explain research in visual cognition to the broader world. When you start with a problem in the world, it is easier to describe your research goals to the person in the next seat on the airplane or to your political representative.
